# Implications of free Shiga toxin-converting bacteriophages occurring outside bacteria for the evolution and the detection of Shiga toxin-producing *Escherichia coli*

**DOI:** 10.3389/fcimb.2014.00046

**Published:** 2014-04-16

**Authors:** Alexandre Martínez-Castillo, Maite Muniesa

**Affiliations:** Department of Microbiology, Faculty of Biology, University of BarcelonaBarcelona, Spain

**Keywords:** bacteriophages, Shiga toxin, STEC, environment, transduction

## Abstract

In this review we highlight recent work that has increased our understanding of the distribution of Shiga toxin-converting phages that can be detected as free phage particles, independently of Shiga toxin-producing bacteria (STEC). Stx phages are a quite diverse group of temperate phages that can be found in their prophage state inserted within the STEC chromosome, but can also be found as phages released from the cell after activation of their lytic cycle. They have been detected in extraintestinal environments such as water polluted with feces from humans or animals, food samples or even in stool samples of healthy individuals. The high persistence of phages to several inactivation conditions makes them suitable candidates for the successful mobilization of *stx* genes, possibly resulting in the genes reaching a new bacterial genomic background by means of transduction, where ultimately they may be expressed, leading to Stx production. Besides the obvious fact that Stx phages circulating between bacteria can be, and probably are, involved in the emergence of new STEC strains, we review here other possible ways in which free Stx phages could interfere with the detection of STEC in a given sample by current laboratory methods and how to avoid such interference.

Shiga toxin-producing *Escherichia coli* (STEC) are important food-borne pathogens and represent a challenge for the scientific community. Effort has been devoted to developing methods for its isolation, detection and identification, to guarantee the quality of products and the health of consumers. Many studies have aimed at gaining an understanding of the mobility of the important gallery of virulence factors that compose the virulome of the *E. coli* pathogenic strains. The mobility of these factors among strains is a key factor for the genotypic and phenotypic variability of this bacterium, and hinders the process of determining what should be considered a true pathogen and how to detect it effectively (Erickson and Doyle, 2007; Karch et al., 2012; Melton-Celsa et al., 2012).

Thus, the methods for *E. coli* detection have focused on those factors that are clearly related with virulence and, particularly, those that are closely related with high incidence and severity of human infections. Among these factors, the Shiga toxin (Stx) is considered one of the most significant and, if not the only one determining pathogenicity, the one that leads to the most undesirable complications of the infection, such as HUS (Tarr et al., 2005).

Stx is a good example of a mobile virulence factor, since the genes encoding for this toxin are located in the genome of temperate bacteriophages (Newland et al., 1985; Huang et al., 1987; O'Brien et al., 1989). Many phages simply multiply by infecting bacteria, killing the host by lysis and releasing new phages. Temperate phages, however, adopt a benign relationship with their hosts, called lysogeny, which allows an attachment of phage DNA to a bacterial chromosome. For Stx phages, once a phage integrates its genome within the bacterial genome, the bacteria acquires the *stx* gene and with it the capacity to express the toxin; thereby becoming a STEC. Temperate phages can revert from the lysogenic state by entering the lytic cycle, mostly due to environmental conditions or exogenous factors. The lytic pathway starts by generating multiple copies of its genome, causing an increase in the Stx produced by the cell during the process (Neely and Friedman, 1998; Wagner et al., 2001; Tyler et al., 2004). By the expression of the phage genome, new phage capsids are generated, assembled with the phage DNA and, once formed, phage particles are released from the cell by lysis. Once released, phage particles remain free outside the bacterial cell, and here is where our story starts.

The major question we address is: what happens to phages outside the cell and to what extent does their occurrence as free particles play a role in the evolution of new STEC strains?

Phages are rather simple particles and Stx phages are no exception. Their persistence outside the cell is guaranteed by their ability to circumvent natural and artificial inactivation processes (as reviewed in the next section), many of which would inactivate their bacterial host. It is the fact that the life cycle of phages switches to the lytic state that allows phages to be released once the cell has been threatened by factors that activate its SOS response (Muhldorfer et al., 1996; Kimmitt et al., 2000; Köhler et al., 2000; Yamamoto et al., 2003; Aertsen et al., 2005; Toshima et al., 2007; Pacheco and Sperandio, 2009).

## Stx phages in fecally polluted waters

Several reports have shown the occurrence of free Stx phages in water environments with fecal pollution or directly on homogenates of fecal samples (Table 1). Most studies have only focused on the detection of Stx phages in general or Stx2 phages in particular, and only a few have evaluated Stx1 phages (Dumke et al., 2006; Yan et al., 2011; Rooks et al., 2012). Those studies show a clear predominance of Stx2 phages over Stx1 phages and, despite the fact that there is still a lack of information on the abundance of Stx1 phages in the environment, the results correlate with reported data on the predominance of stx2 phages over Stx1 phages in lysates induced from environmental *E. coli* isolates (Muniesa et al., 2004a; Garcia-Aljaro et al., 2009; Yan et al., 2011).

**Table 1:** **Occurrence and abundance of free Stx bacteriophages in diverse environments**.

**Sample**	**Country**	**Detection of Stx phages**			**Detection method**	**References**
		**Stx1 phages**	**Stx2 phages**	**Abundance**		
Human wastewater	Spain	–	Positive (I)	10 PFU.ml^−1^	MPN+PCR	Muniesa and Jofre, 1998
Human wastewater	Germany,	–	Positive	≥1 PFU.ml^−1^	PCR	Muniesa and Jofre, 2000
	Austria,		Positive	>0.1PFU.ml^−1^		
	France		Positive	≥10 PFU.ml^−1^		
	Ireland		Positive	≥10 PFU.ml^−1^		
	South Africa		Positive	≥10 PFU.ml^−1^		
	New Zaeland		Positive	≥10 PFU.ml^−1^		
Human wastewater	Japan	–	Positive (I)	–	PCR	Tanji et al., 2003
Human wastewater	Spain	–	Positive (I)	4.24 log_10_ PFU.ml^−1^	Plaque count+PCR	Muniesa et al., 2004b
Cattle wastewater	Spain	–	Positive (I)	4.45 log_10_ PFU.ml^−1^	Plaque count+PCR	Muniesa et al., 2004b
Human wastewater	Germany	Positive (I)	Positive (I)	0.34 PFU.ml^−1^ stx_1_	Plaque count	Dumke et al., 2006
				3.4 PFU.ml^−1^ stx_2_	PCR estimation	
Human treated waste water	Germany	Negative	Positive (I)	–	Plaque count+PCR	Dumke et al., 2006
River water	Germany	Positive (I)	Positive (I)	–	Plaque count+PCR	Dumke et al., 2006
Human wastewater	UK	Negative	Positive (I)	9.41 log_10_ PFU.ml^−1^	Plaque count+PCR	Rooks et al., 2010
Human wastewater	UK	Negative	Positive	2.39 log_10_ GC.ml^−1^	qPCR	Rooks et al., 2010
Human wastewater	Spain	–	Positive	1.37 log_10_ GC.ml^−1^	qPCR	Imamovic et al., 2010
Cattle wastewater	Spain	–	Positive	2.77 log_10_ GC.ml^−1^	qPCR	Imamovic et al., 2010
Pig wastewater	Spain	–	Positive	4.59 log_10_ GC.ml^−1^	qPCR	Imamovic et al., 2010
Poultry wastewater	Spain	–	Positive	1.11 log_10_ GC.ml^−1^	qPCR	Imamovic et al., 2010
Cattle feces	Spain	–	Positive	2.32 log_10_ GC.g^−1^	qPCR	Imamovic et al., 2010
Beef	Spain	–	Positive (I)	4.10 log_10_ GC.g^−1^	qPCR	Imamovic and Muniesa, 2011
Salad	Spain	–	Positive (I)	3.36 log_10_ GC.g^−1^	qPCR	Imamovic and Muniesa, 2011
Swine feces	China	Positive (I)	Positive (I)	–	Plaque count+PCR	Yan et al., 2011
Wastewater	UK	–	Positive (I)	–	Lysogen isolation	Rooks et al., 2012
Human feces	Spain	–	Positive (I)	4.41 log_10_ GC.g^−1^	qPCR	Martinez-Castillo et al., 2013

*GC, gene copy; PFU, plaque forming unit; MPN, most probable number. (I) those studies reporting infectious Stx phages*.

Regardless of the type of toxin variant, the high occurrence of free Stx phages detected suggests that Stx phages could do more than provide intestinal bacteria with a new genetic character. Although a certain level of human fecal contamination is always observed in environments where free Stx phages have been detected (Muniesa and Jofre, 1998; Tanji et al., 2003; Muniesa et al., 2004b; Dumke et al., 2006; Imamovic et al., 2010; Rooks et al., 2010, 2012) (Table 1), there is no total correlation between Stx phage occurrence and fecal pollution, as shown by comparison with fecal indicators (Dumke et al., 2006; Imamovic et al., 2010). This lack of correlation suggests that the fecal origin is the main but not necessarily the sole source of Stx phages. Accordingly, the description of Stx2-positive extraintestinal *E. coli* strains (Wester et al., 2013) supports the suspicion that *stx* is not restricted to a fecal origin. Stx phages are not limited to waters containing human contamination and they have also been found to be highly prevalent when analyzing wastewater from animals (Imamovic et al., 2010; Yan et al., 2011). Water with lower levels of fecal pollution, such as river water, also shows the presence of Stx phages (Muniesa et al., 1999; Dumke et al., 2006).

## Stx phages in feces

If fecally polluted waters present Stx phages, and assuming that Stx phages could have been generated in the intestinal gut, the question that remains to be answered is whether the phages are released into wastewaters by induction from STEC strains present in these environments, or whether free Stx phages are directly excreted through feces. Recent reports indicate that free infectious (those able to infect and propagate in a host strain), Stx2 phages are present in 62% of human feces (Martinez-Castillo et al., 2013). Sequencing of the PCR amplimers suggested that subtypes *stx*_2a_, *stx*_2c_, and *stx*_2d_ were the most frequently detected (Martinez-Castillo et al., 2013). From the positive samples, 90% showed infectious Stx phages. Two facts are to be highlighted from that study: there is not a high incidence of STEC in the area of this study; and the stool samples were taken from healthy individuals without reported gastrointestinal symptoms, and that STEC were not detected in or isolated from these samples.

## Stx phages in food

Assuming that Stx phages are excreted in feces, they must have been ingested, either as free phages or as STEC bacteria that were later induced, once in the intestinal tract. If they are ingested as free phages, then they must be present in food or drinking water, assuming that these are free of significant levels of bacteria. The occurrence of infectious Stx2 phages in commercial samples of beef and salad (Imamovic and Muniesa, 2011), that were acceptable for process hygiene microbiological criteria under EU regulation (Anonymous, 2005), highlights the gap that exists in legislation regarding the presence of phages in food samples.

The fact that phages detected in food were infectious is interesting, considering the likelihood of transduction in food matrices at different pH and temperatures (Imamovic et al., 2009) as well as under dairy processing conditions (Picozzi et al., 2012). This fact is of special relevance considering the observations that many food-processing conditions, such as thermal treatment, high hydrostatic pressures, or UV or other irradiation (Yamamoto et al., 2003; Aertsen et al., 2005; Yue et al., 2012), and the addition of certain compounds during the food production, such as chelating agents (Imamovic and Muniesa, 2011), salt (Harris et al., 2012) or antimicrobials (Kimmitt et al., 2000), not only fail to inactive of the Stx phages, but can enhance Stx phage induction from their STEC hosts.

## Persistence of Stx phages

Generally speaking, phages could persist in different environments or under disinfection processes or inactivation conditions (Dee and Fogleman, 1992; Durán et al., 2002; Jofre, 2007; Lee and Sobsey, 2011). In habitats in which the host bacteria are alien, as fecal bacteria are in the environment, it is likely that phages persist much better than the bacteria (Ogunseitan et al., 1990; Muniesa et al., 1999; Durán et al., 2002). Because of their structural characteristics, their persistence in the environment is high, and these survival capabilities make bacteriophages especially suited for movement and gene transfer between different biomes.

Certain morphologies can be considered more persistent than others, tailed phages being the ones showing higher persistence (Muniesa et al., 1999; Prigent et al., 2005; Lin et al., 2010). Notably, these are the most abundant in different water environments (Prigent et al., 2005; Lin et al., 2010). Stx phages mostly belong to the *Siphoviridae* and *Podoviridae* morphological types (Rietra et al., 1989; Muniesa et al., 2004a,b; Beutin et al., 2012), showing similar persistence to that of other groups of phages (Muniesa et al., 1999; Dumke et al., 2006; Allué-Guardia et al., 2014) and higher persistence than STEC (Muniesa et al., 1999; Mauro and Koudelka, 2011; Rode et al., 2011; Allué-Guardia et al., 2014). Stx phages have been shown to be very stable under food-processing conditions (Yamamoto et al., 2003; Aertsen et al., 2005; Imamovic et al., 2009; Rode et al., 2011; Harris et al., 2012; Yue et al., 2012; Langsrud et al., 2013; Allué-Guardia et al., 2014). In contrast, they do not seem to persist so well under strict treatments such as that linked to the compost model (Johannessen et al., 2005). Nevertheless, their persistence, as for other phages, enhances their mobility between the different environments (feces-food-water) (Mauro and Koudeljka).

Another question is whether the STEC hosts could survive the time necessary to allow phage release before being killed by the treatment applied, or if phages, once released, could remain infectious. Infectivity of Stx phages has been demonstrated after exposure to certain conditions, and some Stx phages remain able to generate lysogens, hence to transduce *stx* (Muniesa and Jofre, 1998; Imamovic et al., 2009; Rode et al., 2011; Yue et al., 2012). The capacity to remain infectious after being subjected to a given condition will determine the chances of *stx* transduction, the real threat in the generation of new STEC strains.

## Interference of Stx phages in STEC detection

The identification of STEC by culture methods in food or clinical samples is advisable to confirm the presence of the pathogen and to further characterize it. However, sometimes strain recovery is not possible because of the low number of STEC cells in a specific sample, the fact that cells could be stressed or in a non-culturable state, or because of the interference of accompanying microbiota, particularly other *E. coli* strains that could confound the detection of the pathogen even when using a specific culture medium. Culture detection of STEC is, in addition, a time-consuming method that hinders early diagnosis of STEC.

Early diagnosis of STEC infection in humans is nevertheless critical for the treatment of the disease, particularly because of the contraindication for treating STEC using antimicrobial agents, and the intense supportive care needed if renal failure occurs (Wong et al., 2000). The need for fast detection and the associated difficulties of STEC isolation from stools in patients treated with antibiotics makes the use of fast and robust molecular methods advisable for STEC detection.

Current laboratory methods for STEC detection, some standardized and approved by national legislation, include PCR-based techniques: either end-point or real-time PCR (Paton and Paton, 2003; Perelle et al., 2004; Gould et al., 2009; Kagkli et al., 2011; Bibbal et al., 2014). Recently, ISO 13136:2012 (Anonymous, 2012), which uses real-time PCR as the reference technology for the detection of the virulence and serogroup-associated genes, has been included in the amended EU regulation for microbiological criteria for sprouts and the sampling rules for poultry carcasses and fresh poultry meat (European commission regulation No. 209/2013). With the same aim in mind, molecular techniques, many based on the information obtained by the use of next-generation sequencing (NGS) methods, are being explored and advised (Baquero and Tobes, 2013; Underwood et al., 2013).

As indicated above, the genomic plasticity of STEC represents a challenge for discriminating pathogenic strains from other non-pathogenic *E. coli* strains present in a given sample. Thus, PCR-based molecular methods are mainly focused on genes related with virulence and on genes that identify a specific serotype, such as *rfb* genes encoding different O antigens (Maurer et al., 1999). Identification of the serotype could be useful for epidemiological purposes and, generally, virulent strains belong to one of the serogroups that account for the non-O157 serotype, including O26, O45, O103, O111, O121, and O145, plus O157:H7 (USDA FSIS, 2012). Lessons learnt from the last outbreak in Germany (Karch et al., 2012) forced researchers to include O104:H4 on the list, and showed that identification of serotype, albeit very useful when treating known pathotypes or for epidemiological purposes, is of limited value for dealing with a newly emerging strain. If STEC isolation is not possible, identifying virulence genes, alone or preferably in combination, appears to be the best approach for assessing the presence of STEC in a sample.

However, when dealing with molecular methods applied to a sample, the presence of free Stx phages, and also other phages that could harbor virulence genes, represents a challenge for the use of PCR-based and NGS methodologies. Moreover, when the protocols include a previous step of selective enrichment of the target bacteria, this step could also maintain or even propagate bacteriophages. This is even more significant if we assume that the methods used for bacterial DNA extraction in these complex matrices will also extract phage DNA (Paton and Paton, 2003; Monday et al., 2007; Grys et al., 2009; ISO 13136:2012). Therefore, any virulence genes present in phage DNA, notably *stx* genes, but also other virulence genes reported in prophages (e.g., *cdt*, *cif*, etc.) (Asakura et al., 2007; Loukiadis et al., 2008) would generate amplimers (PCR) or reads (NGS) that will be interpreted as belonging to STEC, while it could be that they originated in phages. Since in STEC these genes belong to prophages, and therefore are flanked by phage sequences, there is no easy way to distinguish whether the target detected is a bacteria or a phage. If it is a phage, the threat of virulence in humans must be discussed, but it obviously has reduced potential for virulence and would not be enough to raise the alarm.

In addition to those well-characterized phages encoding virulence genes in STEC, many lytic phages could mobilize bacterial genes by generalized transduction. In transduction, after random packaging of bacterial DNA fragments, the genetic material can be mobilized by a phage from a donor bacterium and inserted into a recipient bacterium when it becomes infected by the phage particle. Despite a lack of clear evidence of generalized transduction relating to STEC, any bacterial gene can be mobilized via generalized transduction, including chromosome fragments, though plasmids, transposons and insertion elements, and examples in enterobacteria can also be found (Mann and Slauch, 1997; Schmieger and Schicklmaier, 1999).

Generalized transduction is considered a rare event (Bushman, 2002); however, many approaches for STEC detection based on genomic techniques envisage preliminary steps for the selective or non-selective enrichment of the target microorganism (Hussein and Bollinger, 2008). Enrichment to propagate bacteria can cause the propagation of any sort of phages present in a sample too, a fact that, firstly, would cause a bias in the population of enriched bacteria (Muniesa et al., 2005), and secondly, by increasing the number of phages, would also theoretically cause an increase in the frequency of generalized transduction. As an example to illustrate this point, the protocols for P1 generalized transduction include a propagation step between the donor and the phage to increase the number of generalized transducing particles (Thomason et al., 2007).

Since Shiga toxin is the main virulence threat in STEC, there are many examples of *stx* positive PCR results from samples showing negative detection of culturable STEC. The percentage of samples showing STEC isolates among PCR *stx*-positive samples (either stools of food samples) is in many reports no higher than 50% (Table 2). In clinical stools samples this could be attributable to previous treatment with antimicrobial agents or to a disease being diagnosed late in its course. In food samples it could also be attributable to the presence of non-culturable microorganisms or the presence of bacteria in very low numbers. However, considering the presence of phages as described above, their role in these positive PCR detections or their involvement in sequences generated by NGS cannot be excluded.

**Table 2:** **Frequency of STEC isolation by various methods in samples showing positive result for *stx* (either *stx*_1_ or *stx*_2_) by PCR methods**.

	**No of *stx*-positive samples by PCR**	**No of *stx*-positive samples by PCR with STEC isolation**	**Percentage of PCR *stx*-positive samples with STEC isolation (%)**	**References**
Human stools (healthy slaughterhouse workers)	90	8	8.9	Hong et al., 2009
Humans stools (asymptomatic)	196	47	24	Stephan et al., 2000
Human stools (volunteers)	21	1	4.8	Urdahl et al., 2012
Human stools (hospital)	150	1	0.67	Urdahl et al., 2012
Human stools (hospital)	20^*^	10^*^	50	Buchan et al., 2013
Children stools (hospital)	21	5	24	Vallières et al., 2013
Children stools (hospital)	19	10	52.6	Pradel et al., 2000
Cattle feces	145	80	55.2	Fremaux et al., 2006
Cattle feces	154	67	43.5	Rogerie et al., 2001
Cattle feces	417	18	4.3	Hofer et al., 2012
Cattle feces	330	162	49.0	Pradel et al., 2000
Bovine hides	301	25	8.3	Monaghan et al., 2012
Bovine carcasses	122	5	4.1	Monaghan et al., 2012
Bovine carcasses	77	16	20.8	Breum and Boel, 2010
Bovine carcasses	91	16	17.6	Rogerie et al., 2001
Cattle environment	179	38	21.2	Fremaux et al., 2006
Beef meat	47	16	34.0	Pradel et al., 2000
Dairy buffalo (feces and milk)	56	20	35.7	Beraldo et al., 2014
Milk (bulk)	32	1	3.1	Trevisani et al., 2014
Milk (filters)	68	7	10.3	Trevisani et al., 2014
Cheese	60	5	8.3	Pradel et al., 2000
Healthy pigs feces	255	62	24.3	Meng et al., 2014
Swine feces	484	196	40.5	Fratamico et al., 2004
Meat products	36	8	22.2	Díaz-Sánchez et al., 2012

* *Calculated from the % of positive samples*.

## Avoiding phage interference

The issue that arises is how to avoid phage interference. In theory, several methods could be applied to eliminate phages from the equation. These are mostly based on dismissing the phage population from the sample without interfering with the bacterial population and avoiding complicated steps in the methodology. Some protocols use a centrifugation step after the enrichment culture, and only the pellet containing bacteria is used for DNA extraction (Feng et al., 2011). This method would reduce the number of phages present in the supernatant of the enrichment; though to what extent, has not been tested. Still, phages present in the original samples would have propagated and would still be present in the bacterial pellet.

Filtration has also been proposed to separate phages from the sample and has been widely used to purify phage stocks or phages from a sample before counting (Brock, 1983; EPA, 2000). Microfiltration is mostly applied to retain viruses using the filter and clarify the sample (Van Reis and Zydney, 2001; Saxena et al., 2008), while here the aim is to eliminate phages by passing them through the filter but retaining the bacteria. The selection of the most efficient membrane, in terms of pore size and chemical structure, would be critical with this purpose in mind. The use of 0.22 or 0.45 μm low protein binding membranes that do not retain phages would be advisable. Among these, polyvinylidene fluoride (PVDF), polyethersulfone (PES) or cellulose ester membranes saturated with beef extract have been proposed (Anonymous, 2000; EPA, 2000; Papageorgiou et al., 2000; Mocé-Llivina et al., 2003), and PVDF membranes have been shown to reduce by 2–3 log_10_ the phages present in a bacterial enrichment culture (Muniesa et al., 2005). Complete elimination of all phages present in the samples will probably not be accomplished only by adding a single filtration step, but optimization of this approach would reduce the phage population enough to avoid interference in the molecular detection methods without adding complicated steps to the already-established protocols and without reducing the bacterial population. However, it must be borne in mind that filtration would turn out to be more useful when treating aqueous samples or clear homogenates of solid samples and with a variable efficiency depending on the complexity of the matrix, with lower recoveries expected when it is applied to solid or mixed samples that could clog the membranes.

### Conflict of interest statement

The authors declare that the research was conducted in the absence of any commercial or financial relationships that could be construed as a potential conflict of interest.

## References

[B1] Aertsen A. Faster D. Michiels C. W. (2005). Induction of Shiga toxin-converting prophage in *Escherichia coli* by high hydrostatic pressure. Appl. Environ. Microbiol. 71, 1155–1162 10.1128/AEM.71.3.1155-1162.2005 15746313 PMC1065167

[B2] Allué-Guardia A. Martínez-Castillo A. Muniesa M. (2014). Persistence of infectious Stx bacteriophages after disinfection treatments. Appl. Environ. Microbiol. 80, 2142–2149 10.1128/AEM.04006-13 24463973 PMC3993145

[B3] Anonymous. (2000). ISO 10705-2: Water Quality. Detection and Enumeration of Bacteriophages -Part 2: Enumeration of Somatic Coliphages. Geneva: International Organisation for Standardisation

[B5] Anonymous. (2005). Commission Regulation (EC) No 2073/2005 of 15 November 2005 on microbiological criteria for foodstuffs. Official J. Eur. Commun. 338, 11–25

[B4] Anonymous. (2012). ISO 13136:2012. Microbiology of Food and Animal Feed – Real-time Polymerase Chain Reaction (PCR)-Based Method for the Detection of Food-borne Pathogens – Horizontal Method for the Detection of Shiga Toxin-producing Escherichia coli (STEC) and the Determination of O157, O111, O26, O103 and O145 Serogroups. Geneva: International Organisation for Standardisation

[B6] Asakura M. A. Hinenoya M. S. Alam K. Shima S. H. Zahid L. Shi N. (2007). An inducible lambdoid prophage encoding cytolethal distending toxin (Cdt-I) and a type III effector protein in enteropathogenic *Escherichia coli*. Proc. Natl. Acad. Sci. U.S.A. 104, 14483–14488 10.1073/pnas.0706695104 17726095 PMC1964815

[B7] Baquero F. Tobes R. (2013). Bloody coli: a gene cocktail in *Escherichia coli* O104:H4. MBio 4:e00066-13 10.1128/mBio.00066-13 23422408 PMC3624515

[B8] Beraldo L. G. Borges C. A. Maluta R. P. Cardozo M. V. Rigobelo E. C. de Ávila F. A. (2014). Detection of Shiga toxigenic (STEC) and enteropathogenic (EPEC) *Escherichia coli* in dairy buffalo. Vet. Microbiol. 170, 162–166 10.1016/j.vetmic.2014.01.023 24560591

[B9] Beutin L. Hammerl J. A. Strauch E. Reetz J. Dieckmann R. Kelner-Burgos Y. (2012). Spread of a distinct Stx2-encoding phage prototype among *Escherichia coli* O104:H4 strains from outbreaks in Germany, Norway, and Georgia. J. Virol. 86, 10444–10455 10.1128/JVI.00986-12 22811533 PMC3457275

[B10] Bibbal D. Loukiadis E. Kérourédan M. Peytavin de Garam C. Ferré F. Cartier P. (2014). Intimin gene (eae) subtype-based real-time PCR strategy for specific detection of shiga toxin-producing *Escherichia coli* serotypes O157:H7, O26:H11, O103:H2, O111:H8, and O145:H28 in cattle feces. Appl. Environ. Microbiol. 80, 1177–1184 10.1128/AEM.03161-13 24296503 PMC3911233

[B11] Breum S. O. Boel J. (2010). Prevalence of *Escherichia coli* O157 and verocytotoxin producing *E*. coli(VTEC) on Danish beef carcasses. Int. J. Food Microbiol. 141, 90–96 10.1016/j.ijfoodmicro.2010.03.009 20427097

[B12] Brock T. D. (1983). Membrane Filtration: A User's Guide and Reference Manual. Madison, WI: Science Technology Springer-Verlag

[B13] Buchan B. W. Olson W. J. Pezewski M. Marcon M. J. Novicki T. Uphoff T. S. (2013). Clinical evaluation of a real-time PCR assay for identification of *Salmonella*, *Shigella*, *Campylobacter* (*Campylobacter jejuni* and *C. coli*), and shiga toxin-producing *Escherichia coli* isolates in stool specimens. J. Clin. Microbiol. 51, 4001–4007 10.1128/JCM.02056-13 24048539 PMC3838069

[B14] Bushman F. (2002). Lateral DNA Transfer. New York, NY: Cold Spring Harbor Laboratory

[B15] Dee S. W. Fogleman J. C. (1992). Rates of inactivation of waterborne coliphages by monochloramine. Appl. Environ. Microbiol. 58, 3136–3141 1444427 10.1128/aem.58.9.3136-3141.1992 PMC183060

[B16] Díaz-Sánchez S. Sánchez, S, Sánchez M. Herrera-León S. Hanning I. Vidal D. (2012). Detection and characterization of Shiga toxin-producing *Escherichia coli* in game meat and ready-to-eat meat products. Int. J. Food Microbiol. 160, 179–182 10.1016/j.ijfoodmicro.2012.09.016 23177058

[B17] Dumke R. Schroter-Bobsin U. Jacobs E. Roske I. (2006). Detection of phages carrying the Shiga toxin 1 and 2 genes in waste water and river water samples. Lett. Appl. Microbiol. 42, 48–53 10.1111/j.1472-765X.2005.01809.x 16411919

[B18] Durán A. E. Muniesa M. Méndez X. Valero F. Lucena F. Jofre J. (2002). Removal and inactivation of indicator bacteriophages in fresh waters. J. Appl. Microbiol. 92, 338–347 10.1046/j.1365-2672.2002.01536.x 11849363

[B19] Environmental Protection Agency (EPA). (2000). Method 1602: Male-specific (F+) and Somatic Coliphages in Water by Single Agar Layer (SAL) Procedure. Washington DC: Office of Water. United States Environmental Protection Agency

[B20] Erickson M. C. Doyle M. P. (2007). Food as a vehicle for transmission of Shiga toxin-producing *Escherichia coli*. J. Food Prot. 70, 2426–2449 17969631 10.4315/0362-028x-70.10.2426

[B21] Feng P. Weagant S. D. Jinneman K. (2011). Diarrheagenic *Escherichia coli*, in Inited States Food and Drug Administration. Bacteriological Analytical Manual. Available online at: http://www.fda.gov/Food/FoodScienceResearch/LaboratoryMethods/ucm070080.htm

[B22] Fratamico P. M. Bagi L. K. Bush E. J. Solow B. T. (2004). Prevalence and characterization of shiga toxin-producing *Escherichia coli* in swine feces recovered in the National Animal Health Monitoring System's Swine 2000 study. Appl. Environ. Microbiol. 70, 7173–7178 10.1128/AEM.70.12.7173-7178.2004 15574914 PMC535163

[B23] Fremaux B. Raynaud S. Beutin L. Rozand C.V. (2006). Dissemination and persistence of Shiga toxin-producing *Escherichia coli* (STEC) strains on French dairy ferms. Vet. Microbiol. 117, 180–191 10.1016/j.vetmic.2006.04.030 16837144

[B24] Garcia-Aljaro C. Muniesa M. Jofre J. Blanch A. R. (2009). Genotypic and phenotypic diversity among induced, stx2-carrying bacteriophages from environmental *Escherichia coli* strains. Appl. Environ. Microbiol. 75, 329–336 10.1128/AEM.01367-08 19011056 PMC2620696

[B25] Gould L. H. Bopp C. Strockbine N. Atkinson R. Baselski V. Body B. (2009). Recommendations for diagnosis of shiga toxin–producing *Escherichia coli* infections by clinical laboratories. MMWR Recomm. Rep. 58, 1–14 19834454

[B26] Grys T. E. Sloan L. M. Rosenblatt J. E. Patel R. (2009). Rapid and sensitive detection of Shiga toxin-producing *Escherichia coli* from non-enriched stool specimens by real-time PCR in comparison to enzyme immunoassay and culture. J. Clin. Microbiol. 47, 2008–2012 10.1128/JCM.02013-08 19439539 PMC2708480

[B27] Harris S. M. Yue W. F. Olsen S. A. Hu J. Means W. J. McCormick R. J. (2012). Salt at concentrations relevant to meat processing enhances Shiga toxin 2 production in *Escherichia coli* O157:H7. Int. J. Food Microbiol. 159, 186–192 10.1016/j.ijfoodmicro.2012.09.007 23107496

[B28] Hofer E. Stephan R. Reist M. Zweifel C. (2012). Application of a real-time PCR-based system for monitoring of O26, O103, O111, O145 and O157 Shiga toxin-producing *Escherichia coli* in cattle at slaughter. Zoonoses Public Health 59, 408–415 10.1111/j.1863-2378.2012.01468.x 22348425

[B29] Hong S. Oh K. H. Cho S. H. Kim J. C. Park M. S. Lim H. S. (2009). Asymptomatic healthy slaughterhouse workers in South Korea carrying Shiga toxin-producing *Escherichia coli*. FEMS Immunol. Med. Microbiol. 56, 41–47 10.1111/j.1574-695X.2009.00545.x 19309486

[B30] Huang A. Friesen J. Brunton J. L. (1987). Characterization of a bacteriophage that carries the genes for production of Shiga-like toxin 1 in *Escherichia coli*. J. Bacteriol. 169, 4308–4312 3040688 10.1128/jb.169.9.4308-4312.1987 PMC213745

[B31] Hussein H. S. Bollinger L. M. (2008). Influence of selective media on successful detection of Shiga toxin-producing *Escherichia coli* in food, fecal, and environmental samples. Foodborne Pathog. Dis. 5, 227–244 10.1089/fpd.2008.0081 18767974

[B32] Imamovic L. Ballesté E. Jofre J. Muniesa M. (2010). Quantification of Shiga toxin-converting bacteriophages in wastewater and in fecal samples by real-time quantitative PCR. Appl. Environ. Microbiol. 76, 5693–5701 10.1128/AEM.00107-10 20622134 PMC2935055

[B33] Imamovic L. Jofre J. Schmidt H. Serra-Moreno R. Muniesa M. (2009). Phage-mediated Shiga toxin 2 gene transfer in food and water. Appl. Environ. Microbiol. *75*, 1764–1768 10.1128/AEM.02273-08 19168651 PMC2655461

[B34] Imamovic L. Muniesa M. (2011). Quantification and evaluation of infectivity of shiga toxin-encoding bacteriophages in beef and salad. Appl. Environ. Microbiol. 77, 3536–3540 10.1128/AEM.02703-10 21441341 PMC3126450

[B35] Jofre J. (2007). Indicators of waterborne enteric viruses in human viruses in water, in Series Perspectives in Medical Virology, Vol. 17, ed Bosch A. (Amsterdam: Elsevier), 227–249 10.1016/S0168-7069(07)17011-7

[B36] Johannessen G. S. James C. E. Allison H. E. Smith D. L. Saunders J. R. McCarthy A. J. (2005). Survival of a Shiga toxin-encoding bacteriophage in a compost model. FEMS Microbiol. Lett. 245, 369–375 10.1016/j.femsle.2005.03.031 15837394

[B37] Kagkli D. M. Weber T. P. Van den Bulcke M. Folloni S. Tozzoli R. Morabito S. (2011). Application of the modular approach to an in-house validation study of real-time PCR methods for the detection and serogroup determination of verocytotoxigenic *Escherichia coli*. Appl. Environ. Microbiol. 77, 6954–6963 10.1128/AEM.05357-11 21856838 PMC3187101

[B38] Karch H. Denamur E. Dobrindt U. Finlay B. B. Hengge R. Johannes L. (2012). The enemy within us: lessons from the 2011 European *Escherichia coli* O104:H4 outbreak. EMBO Mol. Med. 4, 841–848 10.1002/emmm.201201662 22927122 PMC3491817

[B39] Kimmitt P. T. Harwood C. R. Barer M. R. (2000). Toxin gene expression by Shiga toxin-producing *Escherichia coli*: the role of antibiotics and the bacterial SOS response. Emerg. Infect. Dis. 6, 458–465 10.3201/eid0605.000503 10998375 PMC2627954

[B40] Köhler B. Karch H. Schmidt H. (2000). Antibacterials that are used as growth promoters in animal husbandry can affect the release of Shiga-toxin-2-converting bacteriophages and Shiga toxin 2 from *Escherichia coli* strains. Microbiology 146, 1085–1090 10832635 10.1099/00221287-146-5-1085

[B41] Langsrud S. Heir E. Rode T. M. (2013). Survival of Shiga toxin-producing *Escherichia coli* and Stx bacteriophages in moisture enhanced beef. Meat Sci. . [Epub ahead of print]. 10.1016/j.meatsci.2013.09.022 24134920

[B42] Lee H. S. Sobsey M. D. (2011). Survival of prototype strains of somatic coliphage families in environmental waters and when exposed to UV low-pressure monochromatic radiation or heat. Water Res. 45, 3723–3734 10.1016/j.watres.2011.04.024 21600626

[B43] Lin L. Honh W. Ji X. Han J. Huang L. Wei Y. (2010). Isolation and characterization of an extremely long tail Thermus bacteriophage from Tegchong hot springs in China. J. Basic Microbiol. 50, 452–456 10.1002/jobm.201000116 20806260

[B44] Loukiadis E. Nobe R. Herold S. Tramuta C. Ogura Y. Ooka T. (2008). Distribution, functional expression, and genetic organization of Cif, a phage-encoded type III-secreted effector from enteropathogenic and enterohemorrhagic *Escherichia coli*. J. Bacteriol. 190, 275–285 10.1128/JB.00844-07 17873042 PMC2223761

[B45] Mann B. A. Slauch J. M. (1997). Transduction of low copy numbers of plasmids by bacteriophages by bacteriophage P22. Genetics 146, 447–456 9177996 10.1093/genetics/146.2.447 PMC1207987

[B46] Martinez-Castillo A. Quirós P. Navarro F. Miró E. Muniesa M. (2013). Shiga toxin 2-encoding bacteriophages in human fecal samples from healthy individuals. Appl. Environ. Microbiol. 79, 4862–4868 10.1128/AEM.01158-13 23747705 PMC3754710

[B47] Maurer J. J. Schmidt D. Petrosko P. Sanchez S. Bolton L. Lee M. D. (1999). Development of primers to O-antigen biosynthesis genes for specific detection of *Escherichia coli* O157 by PCR. Appl. Environ. Microbiol. 65, 2954–2960 10388689 10.1128/aem.65.7.2954-2960.1999 PMC91442

[B48] Mauro S. A. Koudelka G. B. (2011). Shiga toxin: expression, distribution, and its role in the environment. Toxins 3, 608–625 10.3390/toxins3060608 22069728 PMC3202840

[B49] Melton-Celsa A. Mohawk K. Teel L. O'Brien A. (2012). Pathogenesis of Shiga-toxin producing *Escherichia coli*. Curr. Top Microbiol. Immunol. 357, 67–103 10.1007/82_2011_176 21915773

[B50] Meng Q. Bai X. Zhao A. Lan R. Du H. Wang T. (2014). Characterization of Shiga toxin- producing *Escherichia coli* isolated from healthy pigs in China. BMC Microbiol. 14:5 10.1186/1471-2180-14-5 24393167 PMC3893481

[B51] Mocé-Llivina L. Jofre J. Muniesa M. (2003). Comparison of polyvinylidene fluoride and polyether sulfone membranes in filtering viral suspensions. J. Virol. Methods. 109, 99–101 10.1016/S0166-0934(03)00046-6 12668275

[B52] Monaghan A. Byrne B. Fanning S. Sweeney T. McDowell D. Bolton D. J. (2012). Serotypes and virulotypes of non-O157 shiga-toxin producing *Escherichia coli* (STEC) on bovine hides and carcasses. Food Microbiol. 32, 223–229 10.1016/j.fm.2012.06.002 22986184

[B53] Monday S. R. Beisaw A. Feng P. C. (2007). Identification of Shiga toxigenic *Escherichia coli* seropathotypes A and B by multiplex PCR. Mol. Cell Probes 21, 308–311 10.1016/j.mcp.2007.02.002 17383154

[B54] Muhldorfer I. Hacker J. Keusch G. T. Acheson D. W. K. Tschaepe H. Kane A. V. (1996). Regulation of the Shiga-like toxin II operon in *Escherichia coli*. Infect. Immun. 64, 495–502 8550198 10.1128/iai.64.2.495-502.1996 PMC173792

[B55] Muniesa M. Blanch A. R. Lucena F. Jofre J. (2005). Bacteriophages may bias outcome of bacterial enrichment cultures. Appl. Environ. Microbiol. 71, 4269–4275 10.1128/AEM.71.8.4269-4275.2005 16085813 PMC1183318

[B56] Muniesa M. Blanco J. E. De Simón M. Serra-Moreno R. Blanch A. R. Jofre J. (2004a). Diversity of stx2 converting bacteriophages induced from Shiga-toxin-producing *Escherichia coli* strains isolated from cattle. Microbiology 150, 2959–2971 10.1099/mic.0.27188-0 15347754

[B96] Muniesa M. Jofre J. (1998). Abundance in sewage of bacteriophages that infect *Escherichia coli* O157:H7 and that carry the Shiga toxin 2 gene. Appl. Environ. Microbiol. 64, 2443–2448 9647813 10.1128/aem.64.7.2443-2448.1998 PMC106409

[B57] Muniesa M. Jofre J. (2000). Occurrence of phages infecting *Escherichia coli* O157H7 carrying the stx 2 gene in sewage from different countries. FEMS Microbiol. Lett. 183, 197–200 10.1111/j.1574-6968.2000.tb08957.x 10650226

[B58] Muniesa M. Lucena F. Jofre J. (1999). Comparative survival of free shiga toxin 2-encoding phages and *Escherichia coli* strains outside the gut. Appl. Environ. Microbiol. 65, 5615–5618 10584029 10.1128/aem.65.12.5615-5618.1999 PMC91769

[B59] Muniesa M. Serra-Moreno R. Jofre J. (2004b). Free Shiga toxin bacteriophages isolated from sewage showed diversity although the *stx* genes appeared conserved. Environ. Microbiol. 6, 716–725 10.1111/j.1462-2920.2004.00604.x 15186350

[B60] Neely M. N. Friedman D. I. (1998). Functional and genetic analysis of regulatory regions of coliphage H-19B: location of shiga-like toxin and lysis genes suggest a role for phage functions in toxin release. Mol. Microbiol. 28, 1255–1267 10.1046/j.1365-2958.1998.00890.x 9680214

[B61] Newland J. W. Strockbine N. A. Miller S. F. O'Brien A. D. Holmes R. K. (1985). Cloning of Shiga-like toxin structural genes from a toxin converting phage of *Escherichia coli*. Science 230, 179–181 10.1126/science.2994228 2994228

[B62] O'Brien A. D. Marques L. R. Kerry C. F. Newland J. W. Holmes R. K. (1989). Shiga-like toxin converting phage of enterohemorrhagic *Escherichia coli* strain 933. Microb. Pathog. 6, 381–390 10.1016/0882-4010(89)90080-6 2671581

[B63] Ogunseitan O. A. Sayler G. S. Miller R. V. (1990). Dynamic interactions of Pseudomonas aeruginosa and bacteriophages in lake water. Microb. Ecol. 19, 171–185 24196310 10.1007/BF02012098

[B64] Pacheco A. R. Sperandio V. (2009). Inter-kingdom signaling: chemical language between bacteria and host. Curr. Opin. Microbiol. 12, 192–198 10.1016/j.mib.2009.01.006 19318290 PMC4852728

[B65] Papageorgiou G. T. Mocé-Llivina L. Christodoulou C. G. Lucena F. Akkelidou D. Ioannou E. (2000). A simple methodological approach for counting and identifying culturable viruses adsorbed to cellulose nitrate membrane filters. Appl. Environ. Microbiol. 66, 194–198 10.1128/AEM.66.1.194-198.2000 10618223 PMC91805

[B66] Paton J. C. Paton A. W. (2003). Methods for detection of STEC in humans: an overview, in Escherichia coli: Shiga Toxin Methods and Protocols. Methods in Molecular Medicine. Vol. 73, eds Philpott D. Ebel F. (Totowa, NJ: Humana Press), 9–26 10.1385/1-59259-316-x:09 12375445

[B67] Perelle S. Dilasser F. Grout J. Fach P. (2004). Detection by 5′-nuclease PCR of Shiga-toxin producing *Escherichia coli* O26, O55, O91, O103, O111, O113, O145 and O157:H7, associated with the world's most frequent clinical cases. Mol. Cell. Probe. 18, 185–192 10.1016/j.mcp.2003.12.004 15135453

[B68] Picozzi C. Volponi G. Vigentini I. Grassi S. Foschino R. (2012). Assessment of transduction of *Escherichia coli* Stx2-encoding phage in dairy process conditions. Int. J. Food Microbiol. 153, 388–394 10.1016/j.ijfoodmicro.2011.11.031 22197444

[B69] Pradel N. Livrelli V. De Champs C. Palcoux J. B. Reynaud A. Scheutz F. (2000). Prevalence and characterization of Shiga toxin-producing *Escherichia coli* isolated from cattle, food, and children during a one-year prospective study in France. J. Clin. Microbiol. 38, 1023–1031 10698990 10.1128/jcm.38.3.1023-1031.2000 PMC86328

[B70] Prigent M. Leroy M. Confalonieri F. Dutertre M. DuBow M. S. (2005). A diversity of bacteriophages forms and genomes can be isolated from the surface sands of Sahara Desert. Extremophiles 9, 289–296 10.1007/s00792-005-0444-5 15947866

[B71] Rietra P. J. Willshaw G. A. Smith H. R. Field A. M. Scotland S. M. Rowe B. (1989). Comparison of Vero-cytotoxin-encoding phages from *Escherichia coli* of human and bovine origin. J. Gen. Microbiol. 135, 2307–2318 10.1099/00221287-135-8-2307 2699332

[B72] Rode T. M. Axelsson L. Granum P. E. Heir E. Holck A. L'abée-Lund T. M. (2011). High stability of Stx2 phage in food and under food-processing conditions. Appl. Environ. Microbiol. 77, 5336–5341 10.1128/AEM.00180-11 21685156 PMC3147457

[B73] Rogerie F. Marecata A. Gambadeb S. Duponda F. Beauboisb P. Langea M. (2001). Characterization of Shiga toxin producing *E*. *coli* and O157 serotype *E*. *coli* isolated in France from healthy domestic cattle. Int. J. Food Microbiol. 63, 217–223 10.1016/S0168-1605(00)00422-0 11246905

[B74] Rooks D. J. Libberton B. Woodward M. J. Allison H. E. McCarthy A. J. (2012). Development and application of a method for the purification of free shigatoxigenic bacteriophage from environmental samples. J. Microbiol. Methods 91, 240–245 10.1016/j.mimet.2012.08.017 22964348

[B75] Rooks D. J. Yan Y. McDonald J. E. Woodward M. J. McCarthy A. J. Allison H. E. (2010). Development and validation of a qPCR-based method for quantifying Shiga toxin-encoding and other lambdoid bacteriophages. Environ. Microbiol. 12, 1194–1204 10.1111/j.1462-2920.2010.02162.x 20148931

[B76] Saxena A. Tripathi B. P. Kumar M. Shahi V. K. (2008). Membrane-based techniques for the separation and purification of proteins: an overview. Adv. Colloid Interface 145, 1–22 10.1016/j.cis.2008.07.004 18774120

[B77] Schmieger H. Schicklmaier P. (1999). Transduction of multiple drug resistance of *Salmonella enterica* serovar Typhimurium DT104. FEMS Microbiol. Lett. 170, 251–256 10.1111/j.1574-6968.1999.tb13381.x 9919675

[B78] Stephan R. Ragettli S. Untermann F. (2000). Prevalence and characteristics of verotoxin-producing *Escherichia coli* (VTEC) in stool samples from asymptomatic human carriers working in the meat processing industry in Switzerland. J. Appl. Microbiol. 88, 335–341 10.1046/j.1365-2672.2000.00965.x 10736003

[B79] Tanji Y. Mizoguchi K. Yoichi M. Morita M. Kijima N. Kator H. (2003). Seasonal change and fate of coliphages infected to *Escherichia coli* O157:H7 in a waste water treatment plant. Water Res. 37, 1136–1142 10.1016/S0043-1354(02)00464-5 12553989

[B80] Tarr P. I. Gordon C. A. Chandler W. L. (2005). Shiga-toxin-producing *Escherichia coli* and haemolytic uraemic syndrome. Lancet 365, 1073–1086 10.1016/S0140-6736(05)71144-2 15781103

[B81] Thomason L. C. Costantino N. Court D. L. (2007). *E. coli* genome manipulation by P1 transduction. Curr. Protoc. Mol. Biol. Chapter 1:Unit 1.17. 10.1002/0471142727.mb0117s79 18265391

[B82] Toshima H. Yoshimura A. Arikawa K. Hidaka A. Ogasawara J. Hase A. (2007). Enhancement of Shiga toxin production in enterohemorrhagic *Escherichia coli* serotype O157:H7 by DNase colicins. Appl. Environ. Microbiol. 73, 7582–7588 10.1128/AEM.01326-07 17933918 PMC2168048

[B83] Trevisani M. Mancusi R. Delle Donne G. Bacci C. Bassi L. (2014). Detection of Shiga toxin (Stx)-producing *Escherichia coli* (STEC) in bovine dairy herds in Northern Italy. Int. J. Food. Microbiol. . [Epub ahead of print]. 10.1016/j.ijfoodmicro.2013.12.033 24495690

[B84] Tyler J. S. Miller M. J. Friedman D. I. (2004). The operator and early promoter region of the Shiga toxin type 2-ecoding bacteriophage 933W and control of toxin expression. J. Bacteriol. 186, 7670–7679 10.1128/JB.186.22.7670-7679.2004 15516581 PMC524894

[B85] Underwood A. P. Dallman T. Thomson N. R. Williams M. Harker K. Perry N. (2013). Public health value of next-generation DNA sequencing of enterohemorrhagic *Escherichia coli* isolates from an outbreak. J. Clin. Microbiol. 51, 232–237 10.1128/JCM.01696-12 23135946 PMC3536255

[B86] Urdahl A. M. Solheim H. T. Vold L. Hasseltvedt V. Wasteson Y. (2012). Shiga toxin-encoding genes (*stx* genes) in human fecal samples. APMIS 121, 202–210 10.1111/j.1600-0463.2012.02957.x 23030833

[B97] USDA FSIS. (2012). Risk Profile for Pathogenic Non-O157 Shiga Toxin-Producing Escherichia coli (non-O157 STEC). Available online at: http://www.fsis.usda.gov/PDF/Non_O157_STEC_Risk_Profile_May2012.pdf

[B87] Vallières E. Saint-Jean M. Rallu F. (2013). Comparison of three different methods for detection of Shiga toxin-producing *Escherichia coli* in a tertiary pediatric care center. J. Clin. Microbiol. 51, 481–486 10.1128/JCM.02219-12 23175264 PMC3553904

[B88] Van Reis R. Zydney A. (2001). Membrane separations in biotechnology. Curr. Opin. Biotechnol. 12, 208–211 10.1016/S0958-1669(00)00201-9 11287239

[B89] Wagner P. L. Neely M. N. Zhang X. Acheson D. W. Waldor M. K. Friedman D. I. (2001). Role for a phage promoter in Shiga toxin 2 expression from a pathogenic *Escherichia coli* strain. J. Bacteriol. 183, 2081–2085 10.1128/JB.183.6.2081-2085.2001 11222608 PMC95105

[B90] Wester A. L. Brandal L. T. Dahle U. R. (2013). Extraintestinal pathogenic *Escherichia coli* carrying the Shiga Toxin gene *stx*_2_. J. Clin. Microbiol. 51, 4279–4280 10.1128/JCM.01349-13 24108605 PMC3838058

[B91] Wong C. S. Jelacic S. Habeeb R. L. Watkins S. L. Tarr P. I. (2000). The risk of the hemolytic-uremic syndrome after antibiotic treatment of *Escherichia coli* O157:H7 infections. N. Engl. J. Med. 342, 1930–1936 10.1056/NEJM200006293422601 10874060 PMC3659814

[B92] Yamamoto T. Kojio S. Taneike I. Nakagawa S. Iwakura N. Wakisaka-Saito N. (2003). ^60^Co irradiation of Shiga toxin (Stx)-producing *Escherichia coli* induces Stx phage. FEMS Microbiol. Lett. 222, 115–121 10.1016/S0378-1097(03)00259-3 12757954

[B93] Yan Y. Shi Y. Cao D. Meng X. Xia L. Sun J. (2011). Prevalence of Stx phages in environments of a pig farm and lysogenic infection of the field *E. coli* O157 isolates with a recombinant converting Phage. Curr. Microbiol. 62, 458–464 10.1007/s00284-010-9729-8 20697714

[B94] Yue W. F. Du M. Zhu M. J. (2012). High temperature in combination with UV irradiation enhances horizontal transfer of *stx_2_* gene from *E. coli* O157:H7 to non-pathogenic *E. coli*. PLoS ONE 7:e31308 10.1371/journal.pone.0031308 22347461 PMC3276539

